# Prognostic Significance of New-Onset Atrial Fibrillation Burden in Acute Myocardial Infarction Patients: A Comparison Based on Left Ventricular Ejection Fraction

**DOI:** 10.3390/jcdd13040169

**Published:** 2026-04-15

**Authors:** Yuan Fang, Xingxu Zhang, Yiwei Zhang, Yiqian Yuan, Xiaoming Qin, Baoxin Liu, Jiachen Luo, Yidong Wei

**Affiliations:** Department of Cardiology, Shanghai Tenth People’s Hospital, Tongji University School of Medicine, Shanghai 200072, China

**Keywords:** myocardial infarction, atrial fibrillation burden, left ventricular ejection fraction, multicenter, retrospective cohort, outcomes

## Abstract

Acute myocardial infarction (AMI) with reduced or preserved left ventricular ejection fraction (LVEF) is associated with distinct prognoses and differing risk factor profiles. However, the use of new-onset atrial fibrillation (NOAF) burden in risk stratification of AMI patients, particularly across LVEF subgroups, remains unclear. We analyzed consecutive AMI patients without prior AF who developed their first in-hospital AF episode between 2014 and 2022. The patients were stratified by LVEF (AMIrEF: <40%; AMIpEF: ≥40%) and AF burden (>10.87% vs. ≤10.87%). The primary endpoint was a major adverse cardiovascular event (MACE), including cardiovascular death and heart failure hospitalization. Among 644 patients with LVEF data, 178 (27.6%) were AMIrEF and 466 (72.4%) were AMIpEF; 248 (38.5%) had a high AF burden. Over a median follow-up time of 4.2 years, the MACE incidence was 18.9 and 23.0 per 100 person-years in low- and high-burden AMIrEF patients, and 7.2 and 17.5 in AMIpEF patients, respectively. After multivariable adjustment, a high NOAF burden was significantly associated with increased MACE in AMIpEF patients [hazard ratio (HR): 2.63, 95% confidence interval (CI): 1.82–3.79], but not in AMIrEF patients [HR: 1.29, 95% CI: 0.79–2.10]. Propensity-matched analysis yielded concordant results [AMIrEF: 1.15 (0.69–1.90); AMIpEF: 2.45 (1.75–3.45)]. In conclusion, a high NOAF burden is strongly associated with adverse long-term cardiovascular outcomes in AMIpEF patients, highlighting its potential utility for risk stratification in this population.

## 1. Introduction

Left ventricular systolic dysfunction (LVSD) is a prevalent complication of acute myocardial infarction (AMI), which primarily results from extensive myocardial necrosis. Left ventricular ejection fraction (LVEF) is the most widely used parameter for assessing left ventricular systolic function and remains a key risk indicator following AMI [1,2]. Previous studies have demonstrated that a reduced LVEF is strongly associated with increased mortality in AMI patients. For instance, a large-scale cohort study involving 70,809 patients reported that a reduced LVEF during the acute phase of AMI was linked to a 1-year post-discharge mortality rate of up to 20% [3]. Moreover, patients with a reduced LVEF are at higher risk of long-term cardiovascular events, including recurrent heart failure (HF) hospitalizations [4,5,6]. Over recent decades, growing evidence has highlighted substantial differences in pathophysiology, clinical characteristics, and prognosis between AMI patients with a reduced LVEF (AMIrEF) and those with a preserved LVEF (AMIpEF) [7]. A nationwide retrospective analysis of 12,988 AMI patients demonstrated that independent predictors of 12-month major adverse cardiovascular events (MACEs) differed according to LVEF strata: prior MI, high Killip stage, three-vessel disease, and renal impairment for those with a ≥40% LVEF, and diabetes mellitus and the use of renin-angiotensin system (RAS) inhibitors for those with a <40% LVEF [8]. These differences highlight the importance of LVEF-specific risk stratification in post-MI survivors. Furthermore, prognostic therapeutic strategies for AMIrEF patients are relatively well established [9,10,11]. AMIpEF patients are often under-recognized in prognostic evaluations due to their generally favorable clinical indicators, which contribute to inadequate risk stratification and, consequently, a lack of definitive treatment recommendations for this group. Therefore, identifying novel prognostic markers in AMIpEF patients is essential to tailoring preventive measures, optimizing individualized treatment, and improving long-term outcomes.

New-onset atrial fibrillation (NOAF) following AMI refers to the occurrence of atrial fibrillation (AF) in patients without a history of AF during the acute phase of AMI [12]. AF burden, defined as the proportion of monitoring time spent in AF, is a crucial clinical indicator for quantitatively describing the occurrence of AF. Previous studies have shown that an increased AF burden is significantly associated with a higher risk of adverse outcomes, including HF and all-cause mortality, among individuals with cardiac implanted electronic devices [13,14]. In the setting of AMI, the NOAF burden has also been identified as a predictor of HF rehospitalization, ischemic stroke, and mortality [12]. Nevertheless, prior studies investigating the relationship between AF burden and clinical outcomes failed to stratify AMI patients by LVEF, thereby overlooking the crucial role of LVEF in this relationship. Consequently, the long-term impact of the NOAF burden across different LVEF categories in AMI patients remains insufficiently understood, limiting its clinical applicability.

Accordingly, leveraging data from the NOAFCAMI-China (New-Onset Atrial Fibrillation Complicating Acute Myocardial Infarction in China; ClinicalTrials.gov, NCT05511649) registry, this study aimed to comprehensively evaluate the prognostic impact of the NOAF burden in AMI patients stratified by LVEF (i.e., AMIrEF and AMIpEF). We further sought to determine its value in risk stratification and clinical decision-making across distinct LVEF subgroups, thereby providing evidence to support more precise and individualized management strategies.

## 2. Materials and Methods

### 2.1. Population

The NOAFCAMI-China is a multicenter, retrospective, observational cohort study conducted in Shanghai Tenth People’s Hospital, The First Affiliated Hospital of Zhengzhou University, and Kaifeng Central Hospital. All eligible patients were required to be adults (18 years or older) and have a confirmed diagnosis of both AMI (with or without ST-segment elevation) and AF in accordance with current guidelines [15]. Exclusion criteria were as follows: (1) prior AF; (2) missing continuous electronic monitoring (CEM) data; (3) rheumatic heart disease; (4) sick sinus syndrome; or (5) undergoing emergent coronary artery bypass grafting surgery (CABG). For the present analysis, we further excluded (1) those without baseline LVEF data (*n* = 44); (2) patients that died during hospital stay (*n* = 74); and (3) patients lost to follow-up (*n* = 50). Ultimately, 644 patients were enrolled in the final analysis. The diagnostic criteria for AMI and ascertainment of NOAF burden have been described previously [12]. All qualified patients were divided into 2 groups based on LVEF. Several previous studies investigating risk factors and outcomes after AMI have used a 40% LVEF cutoff [8,16]. In addition, our prior work showed that the association between NOAF and cardiovascular outcomes was more pronounced in patients with HF with a preserved or mid-range ejection fraction (HFpEF/HFmrEF) [17]. Therefore, the same cutoff was adopted in the present study to evaluate the prognostic impact of the NOAF burden. We defined AMIrEF as the presence of clinical in-hospital AMI when EF was <40% and AMIpEF if EF ≥ 40%. Each group was further stratified into 2 subgroups based on a predefined AF burden cutoff of 10.87%, as previously reported [12]. This study was conducted in accordance with the guidelines of the Declaration of Helsinki and passed the ethical review by the ethics committees of the Shanghai Tenth People’s Hospital (No. SHYS-IEC-5.0/22K238/P01), The First Affiliated Hospital of Zhengzhou University (No. KS-2020-KY-007), and the Kaifeng Central Hospital (No. ky2023013).

### 2.2. Data Collection and Definitions

Baseline characteristics, including demographics, cardiovascular risk factors, medical history, medication use, and in-hospital clinical parameters, were obtained from a detailed review of electronic medical records covering the period up to and including the index hospitalization. AF episodes were identified by on-duty physicians based on CEM recordings, and all AF-related parameters were manually recorded in a centralized electronic database. The primary exposure variable was AF burden (%), defined as the percentage of analyzable CEM time spent in AF (total AF duration/total CEM duration × 100%). The specific calculation method is described in our previously published study [12]. AF onset duration (h), defined as the interval from hospital admission to the first documented AF episode, was recorded for each patient. Fasting blood samples were collected after at least 12 h of fasting to measure routine biochemical indices, including C-reactive protein and other biomarkers. Venous blood samples were collected every 8 h during the first 3 days after admission to dynamically monitor Troponin T (TnT) and N-terminal pro–B-type natriuretic peptide (NT-proBNP) levels. The highest recorded values of NT-pro BNP and TnT during the hospital stay were considered their respective peak levels. Definitions of covariates included in multivariable Cox proportional hazard analysis are displayed in Table S1.

### 2.3. Echocardiography Measurements

Baseline echocardiography was performed within 48 h of admission for the index AMI by experienced experts who were blinded to the patients’ clinical background and management. Left atrial and left ventricular dimensions were assessed in the parasternal long-axis view, while LVEF was calculated using the biplane-modified Simpson method from apical views.

### 2.4. Endpoints and Follow-Up

The primary endpoint was MACE, which were composed of cardiovascular death (CV death) and HF hospitalization. Secondary endpoints included cardiovascular death, HF hospitalization, and ischemic stroke. All deaths without a definite non-cardiovascular cause were classified as cardiovascular deaths. HF hospitalization was diagnosed based on clinical symptoms (e.g., breathlessness, ankle swelling, and fatigue) and signs of elevated jugular venous pressure, or peripheral or pulmonary edema, which required an overnight hospital stay and intravenous diuretic treatment. Ischemic stroke was validated according to radiographic imaging tests, defined as the presence of a new neurologic ischemic deficit with signs or symptoms lasting >24 h. Follow-up data were obtained from medical records and telephone interviews until the occurrence of an outcome of interest, death, or the last follow-up (September 2023), whichever occurred first.

### 2.5. Statistical Analysis

Continuous variables with a normal distribution are presented as the mean ± standard deviation (SD) and were analyzed statistically using independent-sample Student’s t-test; continuous variables with a skewed distribution are presented as the median (interquartile range) and were analyzed using the Mann–Whitney test; qualitative variables are presented as the frequency (percentage) and were analyzed statistically using the χ^2^ or Fisher’s exact test.

For long-term survival analyses, the Kaplan–Meier method was used to estimate event-free survival curves, and differences between groups were compared using the log-rank test. Cox proportional hazards regression models were applied to evaluate the association between NOAF burden and clinical outcomes, with results reported as hazard ratios (HRs) and 95% confidence intervals (CIs). Variables included in the multivariable regression models were chosen based on clinical relevance and previous literature: (1) the Global Registry of Acute Coronary Events (GRACE) score; (2) GRACE risk score, age, sex, MI type, primary percutaneous coronary intervention (PCI) and peak troponin-T; and (3) CHA2DS2-VASc score, as a whole and for its individual components. No missing data were found in the abovementioned covariates. To minimize confounding, propensity score matching (PSM) was performed (Supplemental Method). A restricted cubic spline (RCS) analysis was conducted to explore potential non-linear relationships between the NOAF burden and the aforementioned outcomes in the AMIrEF and AMIpEF groups. All analyses were performed using SPSS (version 27.0.0) and R software (Version 4.2.2).

### 2.6. Sensitivity Analysis

To evaluate the robustness of our findings regarding the dichotomization threshold of AF burden and the stratification of LVEF, the following sensitivity analyses were performed: (1) Median-based grouping: Patients were stratified into a high-burden group (>7.28%) and low-burden group (≤7.28%) using the cohort’s median AF burden of 7.28% as the cutoff; (2) Maximally selected rank statistics: The optimal AF burden threshold of 8.41% was determined using the maxstat package in R, with patients categorized into high-burden (>8.41%) and low-burden (≤8.41%) groups; (3) Literature-derived cutoff: The prognostic stratification threshold of 15.29% from our recent publication was applied to divide patients into high-burden (>15.29%) and low-burden (≤15.29%) groups [18]; and (4) LVEF subgroup analysis: AMIpEF patients were further categorized into those with an LVEF of 40–49% and those with an LVEF ≥50%. The results from all analyses are presented as HRs with 95% CIs. The covariates included in the multivariate regression models are the same as those used in the primary analysis.

## 3. Results

### 3.1. Study Population

**Figure 1:**
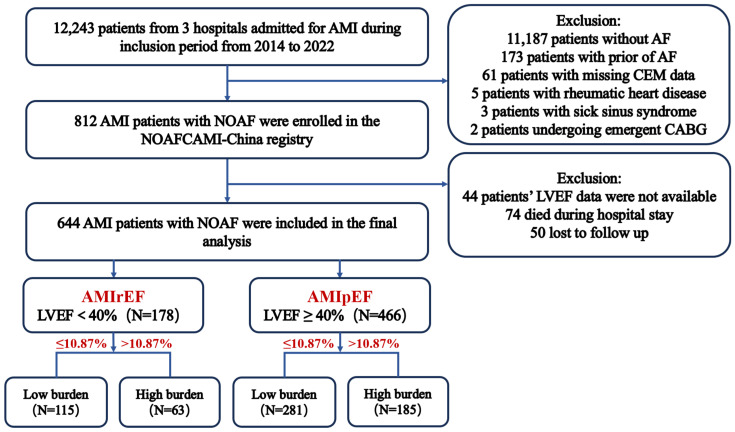
Flow diagram illustrating the inclusion of patients. AF, atrial fibrillation; NOAF, new-onset atrial fibrillation; AMI, acute myocardial infarction; AMIrEF, AMI with reduced ejection fraction; AMIpEF, AMI with preserved ejection fraction; LVEF, left ventricular ejection fraction; CEM, continuous electronic monitoring; CABG, coronary artery bypass grafting.

A total of 812 patients with post-MI NOAF were initially screened, of whom, 168 were excluded due to missing LVEF data or other predefined exclusion criteria. Ultimately, 644 patients were included in the final analysis (mean age: 72.2 ± 11.0 years; 70.0% males) (Figure 1). Compared with the included patients, those excluded exhibited a higher baseline risk profile, characterized by elevated GRACE and CHA_2_DS_2_-VASc scores, a higher Killip class, a faster heart rate, and lower systolic blood pressure (Table S2). Among the included patients, 178 (27.6%) were classified into the AMIrEF group (LVEF < 40%), while 466 (72.4%) were classified into the AMIpEF group (LVEF ≥ 40%). A total of 248 (38.5%) patients had a high AF burden (> 10.87%) during hospitalization. The prevalence of high AF burden was comparable between the AMIrEF and AMIpEF groups (35.4% vs. 39.7%, respectively). The baseline characteristics are presented in Table 1. The patients with a high AF burden had significantly larger baseline left atrial diameters compared to those with a low AF burden. In addition, the patients with a high AF burden were less likely to present with ST-segment elevation myocardial infarction (STEMI), less likely to undergo primary PCI, and had lower peak troponin-T levels. They also exhibited larger left ventricular end-diastolic diameters (LVEDDs) and end-systolic diameters (LVESDs), especially those in the AMIpEF group. Regarding medication management at discharge, the patients with a high AF burden were more frequently prescribed aspirin and oral anticoagulants (Table 2). Furthermore, NOAF-related characteristics indicated that a high AF burden was associated with an earlier onset, longer duration, and lower likelihood of spontaneous termination, whereas the AF episode frequency and symptom prevalence were similar across groups (Table 3).

### 3.2. Long-Term Outcomes

**Figure 2:**
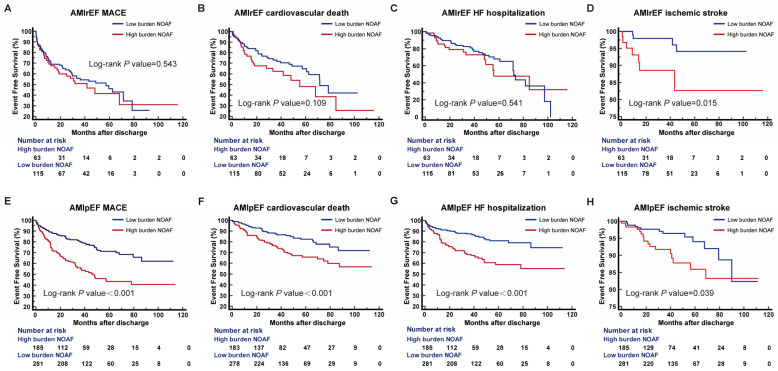
Event-free survival incidences of the studied outcomes in AMIrEF and AMIpEF patients according to AF burden. Kaplan–Meier curves illustrating the event-free survival incidence of MACE (**A**,**E**), cardiovascular death (**B**,**F**), HF hospitalization (**C**,**G**), and ischemic stroke (**D**,**H**) in AMIrEF and AMIpEF patients according to AF burden. NOAF, new-onset atrial fibrillation; AF, atrial fibrillation; AMI, acute myocardial infarction; AMIrEF, AMI with reduced ejection fraction; AMIpEF, AMI with preserved ejection fraction; MACE, major adverse cardiovascular event; HF, heart failure.

Over a median follow-up of 4.2 years (IQR: 2.6–6.2), AMIrEF and AMIpEF patients experienced 86 (48.3%) and 154 (33.0%) MACEs, 64 (35.9%) and 98 (21.0%) CV deaths, 52 (29.2%) and 101 (21.7%) HF hospitalizations, and 11 (6.2%) and 30 (6.4%) ischemic strokes, respectively. The incidence rates of MACE were 18.9 and 23.0 per 100 person-years in the low- and high-burden groups, respectively, among AMIrEF patients, and 7.2 and 17.5 per 100 person-years among AMIpEF patients (Figure 2). Kaplan–Meier analysis demonstrated that a high AF burden was significantly associated with a higher cumulative incidence of all clinical endpoints in AMIpEF patients (all *p*-values from log-rank test < 0.05). In contrast, in the AMIrEF group, no significant differences in MACE or other secondary endpoints were observed between patients with high and low AF burdens, except for ischemic stroke (Figure 2).

In the multivariable Cox regression analysis, a high NOAF burden was significantly associated with a 2.63-fold higher risk of MACE (HR: 2.63, 95% CI: 1.82–3.79; *p* < 0.001) in the AMIpEF group. Similarly, a high AF burden was independently associated with an increased risk of cardiovascular death (HR: 2.01, 95% CI: 1.24–3.26; *p* = 0.004) and HF hospitalization (HR: 2.48, 95% CI: 1.61–3.83; *p* < 0.001) after adjusting for confounders (Table 4). In contrast, no significant associations were observed between AF burden and these outcomes in AMIrEF patients after all adjustments (Table 4). These findings were further confirmed in the propensity score-matched cohort. The baseline characteristics were generally balanced between the groups (Tables S3 and S4). The risk of MACE remained significantly elevated in AMIpEF patients with a high AF burden (HR: 2.45, 95% CI: 1.75–3.45), but not in AMIrEF patients (HR: 1.15, 95% CI: 0.69–1.90), with a similarly elevated risk observed for cardiovascular mortality and HF hospitalization in the AMIpEF subgroup.

Meanwhile, after adjustment for individual components of the CHA_2_DS_2_-VASc score, a high AF burden was significantly associated with an increased risk of ischemic stroke in both the AMIrEF (HR: 3.92, 95% CI: 1.18–13.02; *p* = 0.026) and AMIpEF (HR: 2.08, 95% CI: 1.00–4.32; *p* = 0.049) groups. However, this association was no longer statistically significant in the propensity-matched analysis.

**Figure 3:**
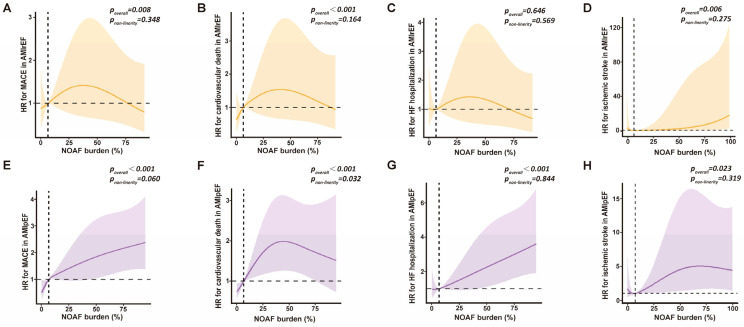
Association between post-MI NOAF burden and clinical outcomes in AMIrEF and AMIpEF groups. RCS analysis of the association between post-MI NOAF burden and risks of MACE, cardiovascular death, HF hospitalization, and ischemic stroke in the AMIrEF (**A**–**D**) and AMIpEF (**E**–**H**) groups. The colored solid line represents the HR, the colored area represents the 95% CI, and the horizontal dashed line indicates an HR of 1.00. For MACE, cardiovascular death, and HF hospitalization, the HRs were adjusted for GRACE risk score, age, sex, MI types, primary PCI, and peak troponin-T. For ischemic stroke, an adjustment was performed using the CHA_2_DS_2_-VASc score. NOAF, new-onset atrial fibrillation; AMI, acute myocardial infarction; AMIrEF, AMI with reduced ejection fraction; AMIpEF, AMI with preserved ejection fraction; MACE, major adverse cardiovascular event; HF, heart failure; RCS, restricted cubic spline; HR, hazard ratio; CI, confidence interval.

Restricted cubic spline analysis revealed that the association between NOAF burden and most outcomes was approximately linear, with a higher AF burden corresponding to a higher risk. An apparent non-linear relationship, with an inverted-U shape, between NOAF burden and cardiovascular death was only observed in the AMIpEF group (Figure 3).

### 3.3. Subgroup and Sensitivity Analysis

**Figure 4:**
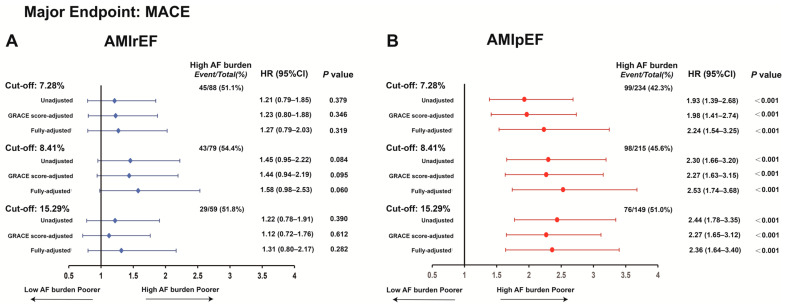
Sensitivity analyses of multiple AF burden thresholds in AMIrEF and AMIpEF patients for the MACE endpoint. Sensitivity analyses evaluating the association between post-MI NOAF burden and MACE using different AF burden cutoffs in AMIrEF (**A**) and AMIpEF (**B**) patients. NOAF, new-onset atrial fibrillation; AF, atrial fibrillation; MI, myocardial infarction; AMI, acute myocardial infarction; AMIrEF, AMI with reduced ejection fraction; AMIpEF, AMI with preserved ejection fraction; MACE, major adverse cardiovascular event; HR, hazard ratio; CI, confidence interval. ^1^ Adjusted for GRACE risk score, age, sex, MI type, primary PCI, and peak troponin-T.

Sensitivity analyses using alternative AF burden thresholds yielded results consistent with the primary analysis. In AMIpEF patients, a high AF burden remained significantly associated with an increased risk of MACE across all cutoff values: HR of 2.24 (95% CI 1.54–3.25) at the 7.28% cutoff, 2.53 (1.74–3.68) at 8.41%, and 2.36 (1.64–3.40) at the 15.29% cutoff, whereas no significant associations were observed in AMIrEF patients (Figure 4).

Further subgroup analysis within the AMIpEF cohort demonstrated that the association between high AF burden and MACE remained significant in both the 40–49% and ≥50% LVEF subgroups, confirming the robustness of the primary findings (Table S5).

## 4. Discussion

The principal findings of this multicenter retrospective study are as follows: (1) In the AMIpEF group, patients with a high AF burden had a 2.63-fold higher risk of MACE than those with a low AF burden. The risks of cardiovascular death and HF hospitalization in high-burden patients were 2.01 and 2.48 times higher, respectively. (2) In the AMIrEF group, the post-MI NOAF burden was not associated with MACE, nor with cardiovascular death or HF hospitalization. This is the first study to comprehensively assess the prognostic significance of the NOAF burden in AMI patients with a specific focus on different LVEF subgroups.

Previous studies have shown that prognostic determinants differ substantially between AMI patients with reduced and preserved LVEFs. These factors include a history of MI, a high Killip stage, three-vessel disease, reduced renal function, and an elevated admission heart rate for AMI patients with an LVEF ≥ 40%, while diabetes mellitus and the use of RAS blockers are predictors for those with an LVEF < 40% [8,16,19]. Identifying these differences is crucial for developing personalized treatment strategies. While prognostic therapeutic strategies for patients with a reduced LVEF are relatively well-defined [9,10,20,21,22], those with a preserved LVEF remain insufficiently characterized in terms of risk stratification and targeted management. Previous studies have demonstrated that AF was significantly associated with long-term outcomes in HF patients, and this association may vary by LVEF category. For example, data from the ESC-HFA HF Long-Term Registry (European Society of Cardiology Heart Failure Long-Term Registry) showed that AF was significantly associated with the composite outcome of all-cause death and HF hospitalization in patients with HFpEF/HFmrEF, but not in those with HF with a reduced ejection fraction (HFrEF) [23]. Similar patterns were observed in other studies [24,25,26]. However, these studies focused solely on the presence of AF rather than quantifying its burden. Given that AF is a progressive disease, its burden—as an indicator of disease progression—may more accurately reflect the associated risk of adverse cardiovascular outcomes [27]. Piccini et al. reported that a weekly increase in AF burden was associated with an increased odds of death [28]. Patients with high-burden subclinical AF had a higher risk of composite adverse outcomes, particularly ischemic stroke and progression to clinical AF, compared with those with a low burden [29]. Moreover, a previous study found that baseline AF burden was only associated with HF events in HFpEF and HFmrEF patients [30], a finding that closely aligns with our results.

Nevertheless, research on the impact of AF burden, a relatively novel AF metric, on the interaction between AF and LVEF, particularly in the context of AMI, remains limited. In a prior sub-study of NOAFCAMI-SH, Hao et al. found that NOAF burden was associated with HF rehospitalization exclusively in HFpEF (HR 1.25, 95% CI: 1.11–1.40) and effectively predicted the risk of HF rehospitalization in this subgroup [17]. However, those findings were based on a small sample from a single-center study. Our current study, using a multicenter cohort of post-MI NOAF patients with longer follow-ups, confirms that AF burden is associated with different prognoses across different LVEF levels.

Specifically, our findings extend the existing evidence by identifying AF burden as a clinically relevant marker that may help refine prognostic assessment specifically in AMIpEF patients—a finding consistent with a retrospective analysis of 1906 patients with AF and HF [31]. In that study, a greater AF burden was significantly associated with a higher risk of adverse outcomes in the subgroup of HF patients with an LVEF ≥ 50% but not in those with an LVEF < 50%. They attributed this difference to the use of β-blockers, postulating that patients treated with β-blockers represented the sickest patients in their analysis. However, no significant difference in β-blocker use was observed in our study. Therefore, the differential prognostic impact of AF burden across LVEF categories observed here may be explained by distinct underlying pathophysiological mechanisms. In AMIrEF patients, disease progression is primarily driven by impaired systolic function, ventricular remodeling, persistent congestion, and low perfusion. Under these circumstances, the incremental contribution of AF burden to overall risk may be relatively limited, as major adverse events are largely dominated by ventricular dysfunction. In contrast, patients with a normal LVEF are more likely to exhibit diastolic dysfunction in which atrial function plays a critical role in maintaining adequate ventricular filling and cardiac output. An increased AF burden may disrupt atrial contraction, impair ventricular filling, and exacerbate hemodynamic instability, thereby directly contributing to adverse cardiovascular outcomes. This mechanism may explain why AF burden demonstrates stronger prognostic significance in AMIpEF patients. However, further studies incorporating direct hemodynamic assessments—such as left atrial strain and diastolic function parameters—are warranted to validate the above hypothesis.

The key clinical implication of this study lies in the fact that AMIpEF patients are often perceived as having a relatively favorable prognosis, which may lead to under-recognition of their long-term risk. Our findings highlight that AF burden, as a critical predictor of adverse cardiovascular outcomes, should be routinely assessed and incorporated into the risk stratification for AMIpEF patients. The subgroup analysis comparing AMIpEF patients with an LVEF of 40–49% versus those with an LVEF ≥ 50% further clarifies the prognostic impact of AF burden, with the burden remaining predictive of MACE in both subgroups. According to current chronic HF classification, an LVEF of 40–49% defines HFmrEF—a clinical entity that lies between HFrEF and HFpEF in some respects but more closely resembles HFrEF in others [32]. Analogous to this intermediate profile, the association was attenuated in the 40–49% LVEF group compared with the ≥ 50% LVEF group. Notably, previous studies seem to suggest that the impact of AF on ischemic stroke is unrelated to LVEF [25,33], which is consistent with our findings. Although AF burden was associated with ischemic stroke in the adjusted analyses, this relationship was not confirmed after propensity score matching. This discrepancy may be explained by the relatively low incidence of stroke events and the reduced statistical power following matching. The low rate of oral anticoagulant use in our cohort may also have masked a potential link between AF burden and stroke risk. Therefore, the relationship between AF burden and stroke risk should be interpreted with caution and requires further investigation. The robustness of our findings is supported by multiple sensitivity analyses using different AF burden thresholds, all of which yielded consistent results. Notably, the cutoff value of 10.87% demonstrated the strongest association with MACE in AMIpEF patients, suggesting its potential utility as a clinically relevant threshold; however, external validation in independent cohorts is warranted to confirm its generalizability.

Although our study was not designed to evaluate therapeutic interventions, the near-linear relationship between AF burden and MACE suggested by our RCS analysis indicates that strategies aimed at reducing AF burden may potentially improve outcomes in this population. Strategies such as optimizing rhythm or rate control, or, in selected cases, catheter ablation—which has been associated with reduced AF burdens and improved outcomes in other populations [34,35]—could be considered for future investigation. Whether such approaches confer prognostic benefit specifically in AMIpEF patients warrants further study.

Finally, several methodological considerations should be taken into account when interpreting our findings. Notably, patients excluded from this analysis had a higher baseline risk profile—including higher GRACE and CHA_2_DS_2_-VASc scores, a higher Killip class, a faster heart rate, and lower systolic blood pressure—than those included. Given that nearly half of the excluded patients died during hospitalization, this pattern was expected. Although these exclusions were necessary to ensure reliable grouping and outcome assessment, they may have introduced selection bias. Consequently, the prognostic value of AF burden in higher-risk patients who died early or lacked key LVEF data remains unclear. Therefore, our findings should be generalized with caution to the broader post-MI population with NOAF. In addition, in this study, AF burden was assessed solely based on in-hospital continuous rhythm monitoring, without follow-up of post-discharge AF status. Consequently, we were unable to capture the dynamic profile of AF burden after MI, particularly the occurrence of AF after discharge and its relationship with long-term outcomes. This limitation inevitably constrains the interpretation of our findings, as cardiovascular outcomes are likely correlated with post-discharge AF status—a dimension that remains beyond the scope of our observational study. Future studies integrating long-term rhythm monitoring devices (e.g., Holter devices, implantable loop recorders, or wearable devices) are warranted to elucidate the respective contributions of acute-phase and post-discharge AF burden to long-term prognosis after MI.

### Limitation

It should be noted that our study has several limitations. First, the retrospective nature of the study limits the ability to infer causality between AF burden and outcomes. Although we adjusted for a variety of baseline covariates, there may still be unmeasured confounders—such as heart rate variability (HRV)—that have an impact on long-term outcomes. In addition, treatment modifications during the clinical course—such as switching from ticagrelor to clopidogrel after the development of NOAF—could not be captured in our retrospective analysis. Second, important atrial structural parameters (e.g., indexed left atrial volume) were not comprehensively collected, and their relationship with prognosis in post-MI NOAF patients requires further study. Third, although our classification strategy follows established methodologies [36,37], we acknowledge that it cannot completely eliminate misclassification bias caused by myocardial stunning. Finally, in this study, AF burden was derived solely from continuous in-hospital electronic monitoring, and we did not systematically capture post-discharge AF status. Therefore, we are unable to determine whether AF persisted after discharge, whether it converted to a sinus rhythm, or the timing of such conversion, nor can we assess how these factors might relate to long-term outcomes.

## 5. Conclusions

A greater NOAF burden was independently associated with increased long-term risk of cardiovascular outcomes in AMIpEF patients but not in those with AMIrEF. Regarding ischemic stroke, the association was less robust and should be interpreted with caution. Our results emphasize the clinical importance of NOAF burden in the risk stratification of AMIpEF patients. Future studies should focus on validating the usefulness of the NOAF burden in this specific population to minimize the risk of MACE.

## Figures and Tables

**Table 1:** Baseline characteristics of participants.

	AMIrEF AMI with LVEF < 40% (*n* = 178)			AMIpEF AMI with LVEF ≥ 40% (*n* = 466)		
	Low AF Burden (*n* = 115)	High AF Burden (*n* = 63)	*p*-Value	Low AF Burden (*n* = 281)	High AF Burden (*n* = 185)	*p*-Value
Demography and medical history						
Age, years	72.3 ± 9.6	73.9 ± 11.1	0.326	71.5 ± 11.1	72.6 ± 11.6	0.366
Gender, men	89 (77.4)	45 (71.4)	0.378	197 (70.1)	120 (65.2)	0.268
Body mass index (kg/m^2^)	24.4 ± 3.6	24.6 ± 4.3	0.822	24.1 ± 3.5	24.6 ± 3.9	0.237
Current smoker	50 (43.5)	23 (36.5)	0.444	99 (35.2)	53 (28.8)	0.316
Hypertension	74 (64.3)	40 (63.5)	0.909	189 (67.3)	138 (75.0)	0.074
Diabetes mellitus	42 (36.5)	33 (52.4)	0.056	109 (38.8)	64 (34.8)	0.382
Dyslipidemia	27 (23.5)	15 (23.8)	0.960	50 (17.8)	35 (19.0)	0.738
Chronic kidney disease	15 (13.0)	8 (12.7)	0.948	20 (7.1)	13 (7.1)	0.983
History of MI	21 (18.3)	8 (12.7)	0.337	17 (6.0)	14 (7.6)	0.510
History of HF	50 (43.5)	28 (44.4)	0.901	32 (11.4)	24 (13.0)	0.592
History of PCI	21 (18.3)	6 (9.5)	0.120	31 (11.0)	18 (9.8)	0.668
Prior stroke	24 (20.9)	10 (15.9)	0.417	48 (17.1)	46 (25.0)	0.050
Prior vascular disease	26 (22.6)	10 (15.9)	0.285	37 (13.2)	26 (14.1)	0.784
Initial presentation						
Out-of-hospital cardiac arrest	2 (1.7)	0 (0)	0.540	9 (3.2)	4 (2.2)	0.711
MI types (STEMI)	80 (69.6)	41 (65.1)	0.540	169 (60.1)	84 (45.7)	0.002
MI location			0.182			0.334
Anterior infarction	62 (78.5)	26 (63.4)		67 (39.6)	34 (41.0)	
Inferior infarction	15 (19.0)	14 (34.1)		95 (56.2)	42 (50.6)	
Other	2 (2.5)	1 (2.4)		7 (4.1)	7 (8.4)	
Killip classes II–IV	42 (36.5)	30 (47.6)	0.149	77 (27.3)	56 (30.4)	0.479
Heart rate (b.p.m.)	97.8 ± 24.7	96.9 ± 32.4	0.842	84.0 ± 19.9	83.5 ± 24.2	0.804
SBP (mmHg)	130.5 ± 23.7	130.0 ± 26.7	0.901	132.9 ± 27.5	137.0 ± 25.9	0.094
GRACE risk score	146.0 ± 29.3	151.6 ± 28.0	0.216	139.2 ± 26.9	140.1 ± 29.6	0.760
CHA2DS2-VASc score	3.7 ± 1.7	3.8 ± 1.8	0.883	3.2 ± 1.7	3.6 ± 1.8	0.021

Comparison of baseline characteristics between low- and high-AF-burden groups in AMIrEF and AMIpEF patients, including demographic features, medical history, and presentation at admission. Values are presented as mean ± SD, median (interquartile range), or *n* (%). MI, myocardial infarction; AMI, acute myocardial infarction; AMIrEF, AMI with reduced ejection fraction; AMIpEF, AMI with preserved ejection fraction; LVEF, left ventricular ejection fraction; AF, atrial fibrillation; HF, heart failure; GRACE, Global Registry of Acute Coronary Events; PCI, percutaneous coronary intervention; SBP, systolic blood pressure.

**Table 2:** Medications and in-hospital examination.

	AMIrEF AMI with LVEF < 40% (*n* = 178)			AMIpEF AMI with LVEF ≥ 40% (*n* = 466)		
	Low AF Burden (*n* = 115)	High AF Burden (*n* = 63)	*p*-Value	Low AF Burden (*n* = 281)	High AF Burden (*n* = 185)	*p*-Value
In-hospital examination						
Creatinine (mg/dL)	86.1 (74.0, 118.3)	95.6 (77.6, 127.4)	0.240	82.5 (68.1, 112.4)	84.9 (70.2, 107.0)	0.694
Peak troponin-T (ng/mL)	4.67 (1.36, 9.47)	3.63 (0.73, 9.78)	0.249	3.39 (1.19, 8.12)	2.03 (0.58, 6.90)	0.045
Lg peak NT-proBNP (pg/mL)	3.88 (3.56, 4.14)	3.91 (3.57, 4.34)	0.531	3.50 (3.06, 3.84)	3.49 (3.11, 3.87)	0.844
eGFR (mL/min)	70.6 (48.7, 87.8)	63.5 (45.1, 84.6)	0.175	77.9 (53.2, 90.2)	71.3 (51.6, 90.9)	0.740
Angiographic data						
Primary PCI	83 (72.2)	46 (73.0)	0.915	226 (80.4)	125 (67.5)	0.002
Infarct-related artery			0.420			0.863
Left anterior descending	65 (61.9)	30 (58.8)		98 (37.0)	60 (39.5)	
Right coronary artery	22 (21.0)	15 (29.4)		105 (39.6)	59 (38.8)	
Left circumflex	18 (17.1)	6 (11.8)		62 (23.4)	33 (21.7)	
Left main disease	16 (13.9)	2 (3.2)	0.029	26 (9.3)	16 (8.7)	0.877
Echocardiographic data						
Left atrial diameter (mm)	39.4 ± 5.7	43.2 ± 5.3	<0.001	38.3 ± 4.8	42.0 ± 5.1	<0.001
LVEDD (mm)	48.0 (45.0, 55.0)	50.5 (44.5, 54.5)	0.603	45.0 (42.0, 48.0)	47.0 (43.5, 51.0)	<0.001
LVESD (mm)	36.5 (32.0, 43.0)	41.0 (33.0, 47.0)	0.216	30.0 (27.0, 33.0)	32.0 (29.0, 37.5)	<0.001
LVEF (%)	33.0 (28.3, 36.0)	33.0 (30.0, 35.0)	0.815	54.0 (45.0, 60.0)	54.0 (45.5, 60.0)	0.614
In-hospital medications						
Aspirin	111 (96.5)	60 (95.2)	0.674	268 (95.4)	171 (92.9)	0.263
Clopidogrel	77 (67.0)	52 (82.5)	0.026	187 (66.5)	130 (70.7)	0.353
Ticagrelor	52 (45.2)	16 (25.4)	0.009	125 (44.5)	66 (35.9)	0.065
Oral anticoagulant	8 (7.0)	14 (22.2)	0.003	16 (5.7)	24 (13.0)	0.006
Statin	111 (96.5)	61 (96.8)	0.915	275 (97.9)	180 (97.3)	0.934
ACEI/ARB	55 (47.8)	29 (46.0)	0.819	149 (53.0)	108 (58.7)	0.229
β-blocker	101 (87.8)	51 (81.0)	0.214	218 (77.6)	139 (75.1)	0.542
CCB	10 (8.7)	13 (20.6)	0.023	50 (17.8)	35 (19.0)	0.738
Diuretic	91 (79.1)	57 (90.5)	0.053	164 (58.4)	125 (67.6)	0.045
Amiodarone	86 (74.8)	38 (60.3)	0.060	173 (61.6)	98 (53.3)	0.076
Medications at discharge						
Aspirin	102 (88.7)	47 (74.6)	0.025	240 (85.4)	133 (72.3)	<0.001
Clopidogrel	71 (61.7)	47 (74.6)	0.058	172 (61.2)	126 (68.5)	0.110
Ticagrelor	43 (37.4)	12 (19.0)	0.013	97 (34.5)	42 (22.8)	0.007
Oral anticoagulation	9 (7.8)	18 (28.6)	<0.001	18 (6.4)	44 (23.9)	<0.001
Statin	109 (94.8)	58 (92.1)	1.000	267 (95.0)	179 (96.8)	0.365
ACEI/ARB	40 (34.8)	19 (30.2)	0.531	124 (44.1)	90 (48.9)	0.311
β-blocker	91 (79.1)	39 (61.9)	0.020	192 (68.3)	118 (63.8)	0.348
CCB	3 (2.6)	5 (7.9)	0.198	33 (11.7)	25 (13.6)	0.542
Diuretic	49 (42.6)	28 (44.4)	0.744	74 (26.3)	65 (35.3)	0.038
Amiodarone	29 (25.2)	14 (22.2)	0.696	34 (12.1)	39 (21.2)	0.008

Comparison of in-hospital parameters between low- and high-AF-burden groups in AMIrEF and AMIpEF patients, including in-hospital examination, echocardiographic data, and medications during hospitalization and at discharge. Values are presented as median (interquartile range) or n (%). AMI, acute myocardial infarction; AMIrEF, AMI with reduced ejection fraction; AMIpEF, AMI with preserved ejection fraction; LVEF, left ventricular ejection fraction; AF, atrial fibrillation; ACEI, angiotensin-converting-enzyme inhibitor; ARB, angiotensin receptor blocker; CCB, calcium-channel blocker; eGFR, estimated glomerular filtration rate; NT-proBNP, N-terminal pro B-type natriuretic peptide; PCI, percutaneous coronary intervention; LVEDD, left ventricular end-diastolic diameter; LVESD, left ventricular end-systolic diameter.

**Table 3:** NOAF characteristics.

	AMIrEF AMI with LVEF < 40% (*n* = 178)			AMIpEF AMI with LVEF ≥ 40% (*n* = 466)		
	Low AF Burden (*n* = 115)	High AF Burden (*n* = 63)	*p*-Value	Low AF Burden (*n* = 281)	High AF Burden (*n* = 185)	*p*-Value
Total CEM duration (h)	205.0 (154.5, 311.4)	185.4 (139.3, 289.1)	0.171	191.5 (138.0, 265.3)	163.0 (123.4, 237.9)	0.008
Total AF duration (h)	6.2 (1.7, 12.0)	91.3 (41.0, 127.1)	<0.001	5.0 (1.4, 9.5)	63.5 (25.0, 127.1)	<0.001
AF burden (%)	3.6 (0.8, 6.7)	42.3 (23.3, 86.3)	<0.001	2.6 (0.9, 6.1)	35.3 (17.8, 72.4)	<0.001
Longest AF episode duration (h)	5.1 (1.6, 9.6)	65.0 (32.0, 103.0)	<0.001	4.4 (1.3, 9.0)	48.0 (24.0, 95.5)	<0.001
AF onset duration (h)	37.8 (11.8, 71.5)	6.5 (0.2, 43.8)	<0.001	33.4 (4.8, 66.0)	9.3 (0.1, 33.6)	<0.001
AF frequency	1 (1, 3)	1 (1, 3)	0.605	1 (1, 2)	1 (1, 2)	0.249
NOAF symptom	70 (60.9)	31 (49.2)	0.116	145 (51.6)	86 (46.7)	0.305

Comparison of NOAF-related indicators between low- and high-AF-burden groups in AMIrEF and AMIpEF patients, reflecting differences in NOAF characteristics. AF onset duration: time from admission to the first documented AF episode. Values are presented as median (interquartile range) or n (%). AMI, acute myocardial infarction; AMIrEF, AMI with reduced ejection fraction; AMIpEF, AMI with preserved ejection fraction; LVEF, left ventricular ejection fraction; AF, atrial fibrillation; CEM, continuous electronic monitoring; NOAF, new-onset atrial fibrillation.

**Table 4:** Long-term clinical outcomes.

Clinical Outcome	AMIrEF AMI with LVEF < 40%			AMIpEF AMI with LVEF ≥ 40%		
	Low AF Burden (*n* = 115)	High AF Burden (*n* = 63)	*p*-Value	Low AF Burden (*n* = 281)	High AF Burden (*n* = 185)	*p*-Value
*MACE*						
Events (%)	55 (47.8)	31 (49.2)	-	66 (23.5)	88 (47.6)	-
Incidence rate (95% CI)	18.9 (14.7–24.0)	23.0 (16.4–31.1)	-	7.2 (5.7–9.2)	17.5 (14.3–21.2)	-
Unadjusted HR	1.00	1.36 (0.84–2.19)	0.214	1.00	2.72 (1.91–3.89)	<0.001
Adjusted HR ^a^	1.00	1.23 (0.76–1.99)	0.407	1.00	2.92 (2.04–4.18)	<0.001
Adjusted HR ^b^	1.00	1.29 (0.79–2.10)	0.308	1.00	2.63 (1.82–3.79)	<0.001
*C* *ardiovascular death*						
Events (%)	38 (33.0)	26 (41.3)	-	42 (14.9)	56 (30.3)	-
Incidence rate (95% CI)	10.4 (7.6–14.2)	16.6 (11.3–23.5)	-	4.2 (3.1–5.7)	8.8 (6.8–11.3)	-
Unadjusted HR	1.00	1.81 (1.04–3.15)	0.036	1.00	2.35 (1.47–3.75)	<0.001
Adjusted HR ^a^	1.00	1.63 (0.93–2.85)	0.087	1.00	2.32 (1.45–3.72)	<0.001
Adjusted HR ^b^	1.00	1.68 (0.95–2.97)	0.073	1.00	2.01 (1.24–3.26)	0.004
*HF hospitalization*						
Events (%)	34 (29.6)	18 (28.6)	-	42 (14.9)	59 (31.9)	-
Incidence rate (95% CI)	11.7 (8.3–16.1)	13.3 (8.3–20.5)	-	4.6 (3.4–6.2)	11.7 (9.1–14.9)	-
Unadjusted HR	1.00	1.06 (0.56–2.00)	0.870	1.00	2.55 (1.67–3.91)	<0.001
Adjusted HR ^a^	1.00	1.01 (0.53–1.93)	0.972	1.00	2.71 (1.77–4.16)	<0.001
Adjusted HR ^b^	1.00	1.05 (0.55–2.01)	0.892	1.00	2.48 (1.61–3.83)	<0.001
*Ischemic stroke*						
Events (%)	4 (3.5)	7 (11.1)	-	13 (4.6)	17 (9.2)	-
Incidence rate (95% CI)	1.1 (0.3–3.1)	4.7 (2.1–9.8)	-	1.3 (0.7–2.3)	2.9 (1.7–4.6)	-
Unadjusted HR	1.00	3.27 (1.03–10.41)	0.044	1.00	2.11 (1.02–4.34)	0.044
Adjusted HR ^c^	1.00	3.34 (1.05–10.65)	0.042	1.00	2.09 (1.01–4.31)	0.046
Adjusted HR ^d^	1.00	3.92 (1.18–13.02)	0.026	1.00	2.08 (1.00–4.32)	0.049
Propensity score-matched analyses						
	Low AF burden (*n* = 84)	High AF burden (*n* = 52)	*p*-value	Low AF burden (*n* = 253)	High AF burden (*n* = 169)	*p*-value
*MACE*						
2:1 Matching	1.00	1.15 (0.69–1.90)	0.599	1.00	2.45 (1.75–3.45)	<0.001
*C* *ardiovascular death*						
2:1 Matching	1.00	1.33 (0.75–2.36)	0.330	1.00	2.15 (1.41–3.29)	<0.001
*HF hospitalization*						
2:1 Matching	1.00	1.14 (0.59–2.18)	0.704	1.00	2.50 (1.64–3.80)	<0.001
*Ischemic stroke*						
2:1 Matching	1.00	2.99 (0.84–10.68)	0.092	1.00	1.79 (0.85–3.76)	0.128

Comparison of unadjusted and adjusted long-term outcomes (MACE, cardiovascular death, heart failure hospitalization, and ischemic stroke) between low- and high-AF-burden groups in AMIrEF and AMIpEF patients, along with corresponding outcomes after propensity score matching. AMI, acute myocardial infarction; AMIrEF, AMI with reduced ejection fraction; AMIpEF, AMI with preserved ejection fraction; LVEF, left ventricular ejection fraction; AF, atrial fibrillation; HF, heart failure; MACE, major adverse cardiovascular event; HR, hazard ratio; CI, confidence interval ^a^ Adjusted for GRACE risk score. ^b^ Adjusted for GRACE risk score, age, sex, MI type, primary PCI, and peak troponin-T. ^c^ Adjusted for CHA2DS2-VASc score. ^d^ Adjusted for individual components in CHA2DS2-VASc score.

## Data Availability

Data are available upon reasonable request.

## Supplementary Materials

The following supporting information can be downloaded at: https://www.mdpi.com/article/10.3390/jcdd13040169/s1, Table S1: Definitions of baseline covariates; Table S2: Baseline characteristics of excluded and included patients; Table S3: Baseline characteristics of AMIrEF patients with low and high burden NOAF before and after propensity-score matching; Table S4: Baseline characteristics of AMIpEF patients with low and high burden NOAF before and after propensity-score matching; Table S5: Sensitivity Analyses of AMIpEF Subgroups for the MACE Endpoint.

## Abbreviations

The following abbreviations are used in this manuscript:

AF	Atrial fibrillation
AMI	Acute myocardial infarction
AMIpEF	Acute myocardial infarction with preserved ejection fraction
AMIrEF	Acute myocardial infarction with reduced ejection fraction
CA	Catheter ablation
CABG	Coronary artery bypass grafting surgery
CEM	Continuous electronic monitoring
CI	Confidence interval
CV death	Cardiovascular death
GRACE	Global Registry of Acute Coronary Events
HF	Heart failure
HFmrEF	HF with mildly reduced ejection fraction
HFpEF	HF with preserved ejection fraction
HFrEF	HF with reduced ejection fraction
HR	Hazard ratio
LVEDD	Left ventricular end-diastolic diameter
LVEF	Left ventricular ejection fraction
LVESD	Left ventricular end-systolic diameter
LVSD	Left ventricular systolic dysfunction
MACE	Major adverse cardiac events
NOAF	New-onset atrial fibrillation
NOAFCAMI-China	New-onset atrial fibrillation complicating acute myocardial infarction in China
NT-pro BNP	N-terminal pro-B-type natriuretic peptide
PCI	Percutaneous coronary intervention
RAS	Renin–angiotensin system
SD	Standard deviation
STEMI	ST-segment elevation myocardial infarction
TnT	Troponin T
