# Supplementary material for: Prognostic Significance of New-Onset Atrial Fibrillation Burden in Acute Myocardial Infarction Patients: A Comparison Based on Left Ventricular Ejection Fraction
Source: J Cardiovasc Dev Dis. 2026 Apr 15;13(4):169. doi: 10.3390/jcdd13040169 (PMC13115877; doi:10.3390/jcdd13040169)
Supplement: Supplementary file 1 [file jcdd-13-00169-s001.zip › jcdd-4201327-supplementary.pdf]

Supplementary Materials

1. Supplementary Method
2. Supplementary Table S1. Definitions of baseline covariates
3. Supplementary Table S2. Baseline characteristics of excluded and included patients
4. Supplementary Table S3. Baseline characteristics of AMIrEF patients with low and high burden NOAF before and after propensity-score matching
5. Supplementary Table S4. Baseline characteristics of AMIpEF patients with low and high burden NOAF before and after propensity-score matching
6. Supplementary Table S5 Sensitivity Analyses of AMIpEF Subgroups for the MACE Endpoint.

Supplementary Method

Propensity-score matching method

Propensity scores derived from a binary logistic regression analysis were used to balance baseline characteristics in AMIrEF and AMIpEF patients with low or high burden of new-onset atrial fibrillation (NOAF). Patients who had low AF burden or high AF burden were matched 2:1 in random order using the nearest neighbor approach with the MatchIt package in R software (Version 4.2.2), with a caliper width set to 0.10 of the propensity score's SD. Differences between the low and high AF burden groups, both before and after propensity-score matching, were evaluated using standardized mean differences (SMDs), with SMD < 0.2 considered adequate balance. Variables used to calculate the propensity scores included age, sex, current smoker, GRACE risk score, CHA2DS2-VASc score, medical history (hypertension, diabetes, hyperlipidemia, chronic kidney disease, heart failure, myocardial infarction, percutaneous coronary intervention, peripheral artery disease, stroke), and initial presentation (ST-segment elevation myocardial infarction [STEMI], Killip class, systolic blood pressure, heart rate).

Supplemental Table S1. Definitions of baseline covariates

Variables	Definitions
MI types	MI types include STEMI and non-STEMI.
primary PCI	Primary PCI was performed as the first-line treatment in patients with STEMI who present with significant symptoms of myocardial ischemia and show evidence of occlusion of the infarct-related coronary artery. Primary PCI success was defined as the restoration of TIMI 3 blood flow in the culprit vessel, accompanied by symptom relief, ST-segment resolution on the ECG, successful completion of the coronary angiography procedure, and survival to discharge.
Peak TnT (ng/mL)	Blood samples were collected from all patients to measure TnT levels using the Elecsys® Troponin-T high-sensitive assay (Roche Inc.) every 8 hours and on the first, second, and third days after admission in an independent laboratory. The highest recorded TnT value was designated as the peak level.
GRACE risk score	The GRACE risk score incorporates eight prognostic factors: age, heart rate, systolic blood pressure, Killip class, serum creatinine, ST-segment deviation, elevated TnT, and pre-hospital cardiac arrest, calculated using the GRACE 2.0 ACS Risk Calculator.
CHA2DS2-VASc score	The CHA2DS2-VASc score includes the following factors: congestive heart failure, hypertension, age (65-74 years), diabetes, vascular disease, and female sex, each of which is assigned 1 point, while age ≥75 years and a history of stroke/TIA are assigned 2 points.

MI, myocardial infarction; STEMI, ST-segment elevation myocardial infarction; non-STEMI, Non-ST-segment elevation myocardial infarction; PCI, percutaneous coronary intervention; ECG, electrocardiogram; TIMI, Thrombolysis in Myocardial Infarction; GRACE, Global Registry of Acute Coronary Events; TIA, transient ischemic attack.

Supplemental Table S2. Baseline characteristics of excluded and included patients

	**Excluded patients (n=168)**	**Included patients (n=644)**	***p-value***
Age, years	74.6±10.2	72.2±11.0	0.011
Gender, Men	101 (60.1)	451 (70.0)	0.014
Body mass index(kg/m2)	24.00±4.2	24.3±3.7	0.443
Current smoker	46 (27.4)	225 (34.9)	0.056
Hypertension	120 (71.4)	441 (68.5)	0.461
Diabetes mellitus	79 (47.0)	248 (38.5)	0.045
Dyslipidemia	24 (14.3)	127 (19.7)	0.107
Chronic kidney disease	14 (8.3)	56 (8.7)	0.882
History of MI	29 (17.3)	60 (9.3)	0.003
History of HF	37 (22.0)	134 (20.8)	0.731
History of PCI	25 (14.9)	76 (11.8)	0.281
Prior stroke	37 (22.0)	128 (19.9)	0.538
Prior vascular disease	40 (23.8)	99 (15.4)	0.010
MI types (STEMI)	99 (58.9)	374 (58.1)	0.842
Killip class II–IV	88 (52.4)	205 (31.8)	< 0.001
Heart rate (b.p.m.)	94.4±26.0	87.6±24.2	0.001
SBP (mmHg)	125.1±27.5	133.3±26.4	< 0.001
GRACE risk score	159.9±29.7	141.7±29.0	< 0.001
CHA2DS2-VASc score	4.1±1.9	3.5±1.8	< 0.001

Values are presented as mean±SD, or n (%). MI, myocardial infarction; HF, heart failure; PCI, percutaneous coronary intervention; STEMI, ST-segment elevation myocardial infarction; GRACE, global registry of acute coronary events; SBP, systolic blood pressure.

Supplementary Table S3 Baseline characteristics of AMIrEF patients with low and high burden NOAF before and after propensity-score matching

Characteristics	Before matching			After matching		
	Low burden (N=115)	High burden (N=62)	Standardized difference	Low burden (N=84)	High burden (N=52)	Standardized difference
**Demography and medical history**						
Age, years	72.3±9.6	73.9±11.2	0.151	73.8±8.8	72.9±11.0	0.092
Men	89 (77.4)	45 (72.6)	0.111	65 (77.4)	39 (75.0)	0.056
Current smoker	50 (43.5)	23 (37.1)	0.130	37 (44.0)	21 (40.4)	0.074
Hypertension	74 (64.3)	39 (62.9)	0.030	57 (67.9)	33 (63.5)	0.093
Diabetes Mellitus	42 (36.5)	33 (52.4)	0.308	34 (40.5)	23 (44.2)	0.076
Hyperlipidemia	27 (23.5)	14 (22.6)	0.021	14 (16.7)	11 (21.2)	0.115
Chronic kidney disease	15 (13.0)	8 (12.9)	0.004	14 (16.7)	6 (11.5)	0.065
History of heart failure	50 (43.5)	28 (45.2)	0.034	32 (38.1)	22 (42.3)	0.148
Peripheral artery disease	26 (22.6)	10 (16.1)	0.165	16 (19.0)	9 (17.3)	0.045

Prior MI	21 (18.3)	8 (12.9)	0.148	11 (13.1)	8 (15.4)	0.066
Prior PCI	21 (18.3)	6 (9.7)	0.250	11 (13.1)	6 (11.5)	0.047
Prior stroke	24 (20.9)	9 (14.5)	0.167	17 (20.2)	9 (17.3)	0.075
**Initial presentation**						
STEMI	80 (69.6)	41 (66.1)	0.074	56 (66.7)	35 (67.3)	0.014
Killip class>1	42 (36.5)	29 (46.8)	0.209	33 (39.3)	22 (42.3)	0.062
SBP, mmHg	130.4±23.8	130.0±26.9	0.020	130.6±22.6	129.7±24.7	0.037
Heart rate, beats per minute	97.8±24.7	97.0±32.6	0.030	97.9±25.0	99.3±31.6	0.048
GRACE risk score	146.0±29.3	151.7±28.3	0.197	149.7±27.3	148.7±28.1	0.035
CHA_2DS_2-VASc score	3.7±1.7	3.7±1.8	0.002	3.8±1.6	3.7±1.8	0.075

SMD < 0.2 indicates adequate balance between groups. AMI, acute myocardial infarction; AMIrEF, AMI with reduced ejection fraction; NOAF, new-onset atrial fibrillation; GRACE, global registry of acute coronary events; SBP, systolic blood pressure; SR, sinus rhythm; STEMI, ST-segment elevation myocardial infarction; SMD, standardized mean difference.

Supplementary Table S4 Baseline characteristics of AMIpEF patients with low and high burden NOAF before and after propensity-score matching

Characteristics	Before matching			After matching		
	Low burden (N=281)	High burden (N=184)	Standardized difference	Low burden (N=253)	High burden (N=169)	Standardized difference
**Demography and medical history**						
Age, years	71.5 ±11.1	72.7 ±11.8	0.097	71.7±11.1	72.3±11.8	0.053
Men	197 (70.1)	119 (64.7)	0.116	172 (68.0)	112 (66.3)	0.036
Current smoker	99 (35.2)	52 (28.3)	0.178	80 (31.6)	51 (30.2)	0.041
Hypertension	189 (67.3)	137 (74.5)	0.159	177 (70.0)	125 (74.0)	0.089
Diabetes Mellitus	109 (38.8)	63 (34.2)	0.095	93 (36.8)	61 (36.1)	0.014
Hyperlipidemia	50 (17.8)	35 (19.0)	0.032	47 (18.6)	34 (20.1)	0.039
Chronic kidney disease	20 (7.1)	13 (7.1)	0.002	19 (7.5)	10 (5.9)	0.064
History of heart failure	32 (11.4)	24 (13.0)	0.028	29 (11.5)	23(13.6)	0.065
Peripheral artery disease	37 (13.2)	26 (14.1)	0.062	24 (9.5)	18 (10.7)	0.039

Prior MI	17 (6.0)	14 (7.6)	0.062	16 (6.3)	12 (7.1)	0.031
Prior PCI	31 (11.0)	18 (9.8)	0.041	25 (9.9)	18 (10.7)	0.025
Prior stroke	48 (17.1)	46 (25.0)	0.195	46 (18.2)	37 (21.9)	0.093
**Initial presentation**						
STEMI	169 (60.1)	84 (45.7)	0.293	145 (57.3)	83 (49.1)	0.165
Killip class>1	77 (27.4)	56 (30.4)	0.136	72 (28.4)	53 (31.4)	0.094
SBP, mmHg	132.9±27.5	137.0±25.9	0.153	133.9±27.6	136.5±26.2	0.099
Heart rate, beats per minute	84.0±21.0	83.5±22.6	0.025	83.8±20.6	84.1±22.6	0.014
GRACE risk score	139.3±27.0	139.4±31.3	0.004	139.5±26.8	140.1±29.8	0.023
CHA_2DS_2-VASc score	3.2±1.7	3.6±1.8	0.219	3.1±1.8	3.5±1.8	0.128

SMD < 0.2 indicates adequate balance between groups. AMI, acute myocardial infarction; AMIpEF, AMI with preserved ejection fraction; NOAF, new-onset atrial fibrillation; GRACE, global registry of acute coronary events; SBP, systolic blood pressure; SR, sinus rhythm; STEMI, ST-segment elevation myocardial infarction; SMD, standardized mean difference.

Supplementary Table S5 Sensitivity Analyses of AMIpEF Subgroups for the MACE Endpoint.

	AMIpEF (LVEF 40–49%)			AMIpEF (LVEF ≥50%)		
**Clinical outcomes**	Low AF burden (N=94)	High AF burden (N=56)	***p-value***	Low AF burden (N=187)	high AF burden (N=129)	***p-value***
MACE						
Events (%)	28 (29.8)	32 (57.1)	-	38 (20.3)	56 (43.4)	-
Incidence rate (95% CI)	10.4 (7.1-14.8)	21.5 (15.4-29.1)	-	5.9 (4.3-8.1)	15.7 (12.2-20.1)	-
Unadjusted HR	1.00	2.14 (1.23-3.73)	0.008	1.00	3.19 (1.99-5.10)	＜0.001
Adjusted HR[a]	1.00	2.23 (1.28-3.91)	0.005	1.00	3.44 (2.14-5.51)	＜0.001
Adjusted HR[b]	1.00	1.99 (1.08-3.66)	0.027	1.00	3.46 (2.13-5.60)	＜0.001

Sensitivity analyses comparing the association between high AF burden and MACE in AMIpEF patients with LVEF 40–49% and those with LVEF ⩾50%. HRs with 95% CIs are presented for each subgroup. AMI, acute myocardial infarction; AMIpEF, AMI with preserved ejection fraction; LVEF, left ventricular ejection fraction; AF, atrial fibrillation; MACE, major adverse cardiovascular event; HR, hazard ratio; CI, confidence interval.

[a]Adjusted for GRACE risk score.

[b]Adjusted for GRACE risk score, age, sex, MI types, primary PCI, and peak troponin-T.
